# C5a–C5AR1 axis as a potential trigger of the rupture of intracranial aneurysms

**DOI:** 10.1038/s41598-024-53651-7

**Published:** 2024-02-07

**Authors:** Akihiro Okada, Kampei Shimizu, Akitsugu Kawashima, Tomomichi Kayahara, Masahiko Itani, Hiroki Kurita, Susumu Miyamoto, Hiroharu Kataoka, Tomohiro Aoki

**Affiliations:** 1 Department of Molecular Pharmacology, Research Institute, National Cerebral and Cardiovascular Center, Osaka, Japan; 2 Department of Neurosurgery, Kyoto University Graduate School of Medicine, Kyoto, Japan; 3 Core Research for Evolutional Science and Technology from Japan Agency for Medical Research and Development, National Cerebral and Cardiovascular Center, Osaka, Japan; 4 Department of Neurosurgery, Tokyo Women’s Medical University Yachiyo Medical Center, Chiba, Japan; 5 Department of Cerebrovascular Surgery, Saitama Medical University International Medical Center, Saitama, Japan; 6 Department of Pharmacology, The Jikei University School of Medicine, 3-25-8 Nishi-Shinbashi, Minato-ku, Tokyo, 105-8461 Japan; 7 Department of Neurosurgery, National Cerebral and Cardiovascular Center, Osaka, Japan

**Keywords:** Molecular biology, Diseases, Pathogenesis, Risk factors

## Abstract

Recent studies have indicated the involvement of neutrophil-mediated inflammatory responses in the process leading to intracranial aneurysm (IA) rupture. Receptors mediating neutrophil recruitment could thus be therapeutic targets of unruptured IAs. In this study, complement C5a receptor 1 (C5AR1) was picked up as a candidate that may cause neutrophil-dependent inflammation in IA lesions from comprehensive gene expression profile data acquired from rat and human samples. The induction of C5AR1 in IA lesions was confirmed by immunohistochemistry; the up-regulations of C5AR1/C5ar1 stemmed from infiltrated neutrophils, which physiologically express C5AR1/C5ar1, and adventitial fibroblasts that induce C5AR1/C5ar1 in human/rat IA lesions. In in vitro experiments using NIH/3T3, a mouse fibroblast-like cell line, induction of C5ar1 was demonstrated by starvation or pharmacological inhibition of mTOR signaling by Torin1. Immunohistochemistry and an experiment in a cell-free system using recombinant C5 protein and recombinant Plasmin indicated that the ligand of C5AR1, C5a, could be produced through the enzymatic digestion by Plasmin in IA lesions. In conclusion, we have identified a potential contribution of the C5a–C5AR1 axis to neutrophil infiltration as well as inflammatory responses in inflammatory cells and fibroblasts of IA lesions. This cascade may become a therapeutic target to prevent the rupture of IAs.

## Introduction

The prognosis of aneurysmal subarachnoid hemorrhage (SAH) remains poor despite modern technical advancements in medical care, with a mortality rate of 25–50%^1,2^. It is, therefore, mandatory to develop a novel therapeutic strategy to prevent the rupture of intracranial aneurysm (IA).

The recent experimental studies using rodent models^3–5^ have clarified the crucial role of neutrophils in triggering rupture of IAs; i.e. exacerbation of inflammatory responses and tissue destruction by proteinases like matrix metalloproteinase-9. Histological examinations and gene expression analyses of human specimens have also supported the involvement of neutrophils in the pathogenesis of IAs^6,7^. Importantly, neutrophil-including microenvironment can be observed almost specifically at the site of rupture in lesions induced in animals^3,4^. Therefore, factors regulating the migration of neutrophils into lesions could be therapeutic targets to prevent the rupture of IAs. In the present study, we have identified complement C5a receptor 1 (C5AR1) as a candidate factor mediating the migration of neutrophils from the comprehensive gene expression profile data of IA lesions^4,8^ and examined the potential contribution of C5AR1 to the pathogenesis.

## Materials and methods

### Study approval

The use of human samples in the present research was approved by the local ethical committee at Kyoto University Graduate School of Medicine (#R0456) and at National Cerebral and Cardiovascular Center (#M29-050, #R20126, #R20126-1, #20126-2, #20126-3, #20126-4, #21012) where samples were analyzed and Tokyo Women’s Medical University Yachiyo Medical Center (#4106) where samples were prepared with written informed consent from each case. All research was performed in accordance with the Declaration of Helsinki.

All of the following experiments using animals, including animal care and use, complied with the National Institute of Health’s Guide for the Care and Use of Laboratory Animals and also followed the ARRIVE guidelines (https://arriveguidelines.org)) and were approved by the Institutional Animal Care and Use Committee of National Cerebral and Cardiovascular Center (Approval Number #19036, #20003, #20004, #21004, #21015, #22012, #22041) and of The Jikei University School of Medicine (Approval Number #2023-004, #2023-009). Also, all experiments about recombinant DNA experiment were approved by the Institutional Biosafety Committee of National Cerebral and Cardiovascular Center (Approval Number #2019-14, #20-7, #21-7, #22-7).

### Human specimen and immunohistochemistry

Human IA samples and control arterial walls (superficial temporal artery) were dissected during microsurgical clipping of unruptured IAs with the written informed consent. Dissected specimen was fixed in formalin solution and embedded in paraffin. 4-um thick slices were then prepared for immunohistochemical analysis. After deparaffinization and blocking with 3% donkey serum (#AB_2337258, Jackson ImmunoResearch, West Grove, PA, USA.), slices were incubated with primary antibodies followed by incubation with secondary antibodies conjugated with fluorescence dye. Finally, fluorescent images were acquired on a confocal fluorescence microscope system (FV1000 or FV3000, Olympus, Tokyo, Japan).

Primary antibodies used were as follows; rabbit polyclonal anti-CD88 (C5AR1) antibody (#PAB26140, Abnova, Taipei, Taiwan), mouse monoclonal anti-CD31 antibody (clone JC70A, #M0823, Dako, Glostrup, Denmark), mouse monoclonal anti-smooth muscle α-actin antibody (clone 1A4, #14-9760-82, Thermo Fisher Scientific, Waltham, MA, USA), rabbit polyclonal anti-myeloperoxidase antibody (#ab9535, abcam, Cambridge, UK), mouse monoclonal anti-human C5a/C5a des-Arg antibody (clone 2952, #HM2079, Hycult Biotech, Wayne, PA, USA), rabbit polyclonal anti-tPA antibody (#10147-1-AP, Proteintech Group, Rosemont, IL, USA), and rabbit polyclonal anti-plasminogen antibody (#GTX102877, Gene Tex, Irvine, CA, USA).

Secondary antibodies used were as follows; Alexa Fluor 488-conjugated donkey anti-mouse IgG H&L antibody (#A21202, Thermo Fisher Scientific), Alexa Fluor 488-conjugated donkey anti-rabbit IgG H&L antibody (#A21206, Thermo Fisher Scientific), Alexa Fluor 594-conjugated donkey anti-mouse IgG H&L antibody (#A21203, Thermo Fisher Scientific), and Alexa Fluor 594-conjugated donkey anti-rabbit IgG H&L antibody (#A21207, Thermo Fisher Scientific).

### Rodent IA models and histological analysis of induced IA lesions

7 week-old male or 10 week-old female Sprague–Dawley rats were purchased from Japan SLC (Shizuoka, Japan). Animals were maintained on a light/dark cycle of 12 h/12 h, and had a free access to chow and water.

To induce IA, under general anesthesia by the intraperitoneal injection of pentobarbital sodium (50 mg/kg, Somnopentyl, Kyoritsu Seiyaku Corporation, Tokyo, Japan) and/or the inhalation of Isoflurane (induction; 5.0%, maintenance; 1.5–2.0%, #IYESC-0001, Pfizer Inc., New York, NY, USA), 7-week-old male rats were subjected to ligation of the left carotid artery and systemic hypertension, achieved by the combination of a high salt diet and ligation of the left renal artery^9^.

In the present study, we defined rupture-prone IAs as IAs at the anterior or posterior communicating artery induced in the rat model demonstrated previously^3,10^ because only IAs at these locations frequently rupture in the rat model. To induce rupture-prone IAs, 10 week-old female rats were subjected to the bilateral ovariectomy, the ligation of the left carotid artery, the right external carotid artery and the right pterygopalatine artery, and systemic hypertension achieved by the combination of a high salt diet and the ligation of the left renal artery under the general anesthesia as same^3,10^.

In both experimental models of IAs, immediately after surgical manipulations, animals were fed the food containing 8% sodium chloride and 0.12% 3-aminopropionitrile (#A0408, Tokyo Chemical Industry, Tokyo, Japan), an inhibitor of lysyl oxidase that catalyzes the cross-linking of collagen and elastin.

At 2 weeks to induce unruptured IA lesions in male rats or 4 months to induce rupture-prone IA lesions in ovariectomized female animals after above surgical manipulations, animals were deeply anesthetized by intraperitoneal injection of the lethal dose of pentobarbital sodium (200 mg/kg, Somnopentyl, Kyoritsu Seiyaku Corporation) or the inhalation of Isoflurane (5.0%, #IYESC-0001, Pfizer Inc.), and transcardially perfused with 4% paraformaldehyde solution. Also, all the dead animals during the observation period were autopsied to assess the onset of SAH. The bifurcation site of anterior cerebral artery—olfactory artery including the induced IA lesion or induced rupture-prone or ruptured lesion at anterior- or posterior communicating artery was stripped, and serial frozen sections were then made.

### Rodent glomerulonephritis model

The anti-Thy-1.1-induced glomerulonephritis model in rats was created following the previous study^11^. Seven-week-old male Wister rats were purchased from Japan SLC (Shizuoka, Japan). Mouse monoclonal anti-CD90 (Th1.1) antibody (1 mg/kg, clone OX-7, lot. 1505LE05, #CL005LE, CiteAb, Bath, UK) was administered intravenously to the rats. At 1 h after the administration of the Thy-1.1 antibody, animals were sacrificed as described above, and the bilateral kidneys were harvested.

### RNA purification and RNA sequencing analysis

Dissected rupture-prone aneurysm and the remaining circle of Willis from the same animal were grinded and homogenized in liquid nitrogen at the 63rd day after surgical manipulations. The total RNA was isolated from homogenized samples using an Rneasy fibrous tissue mini kit (#74704, QIAGEN, Hilden, Germany) with an on-column Dnase treatment, according to the manufacturer’s instructions. The quantity of each total RNA sample was measured using a NanoDrop (Thermo Fisher Scientific), and its quality was assessed by using the RNA integrity number (RIN) on an Agilent 4200 TapeStation (Agilent, Santa Clara, CA, USA). The libraries obtained from the purified RNA samples (500 ng) were then prepared using a TruSeq stranded mRNA sample preparation kit (#20020595, Illumina, San Diego, CA, USA), for the RNA sequencing analyses. Total RNA sequencing was performed on a NextSeq500 (Illumina). Each read was then mapped to the *Rattus norvegicus* reference genome (Rnor6) using CLC genomics workbench (version 11, QIAGEN). Differential expression analyses were performed using the RNA-Seq tool, one similar to the DESeq and the edgeR package, in CLC genomics workbench. The genes whose expression reached a fold change over 1.5 in aneurysm lesions, compared with that in the remaining circle of Willis, were considered to be over-expressed, respectively.

All the raw data from RNA sequencing analysis was deposited to Gene Expression Omnibus (https://www.ncbi.nlm.nih.gov/geo/) (ID # Data will be deposited after the acceptance).

### Immunohistochemistry

5-μm-thick frozen sections were prepared from dissected IA lesions, from the kidneys of the anti-Thy-1.1-induced glomerulonephritis model prepared as described above, or the spleen of rats. After blocking with 3% donkey serum (#AB_2337258, Jackson ImmunoResearch), slices were incubated with primary antibodies followed by incubation with secondary antibodies conjugated with a fluorescence dye. Finally, fluorescent images were acquired on a confocal fluorescence microscope system (FV1000 or FV3000, Olympus).

Primary antibodies used were as follows; mouse monoclonal anti-CD88 (C5AR1) antibody (clone R63, #sc-53797, Santa Cruz Biotechnology, Dallas, Texas, USA), rabbit polyclonal anti-myeloperoxidase antibody (#ab9535, abcam), mouse monoclonal anti-CD68 antibody (#ab31630, clone ED1, abcam), rabbit monoclonal anti-phospho-S6 ribosomal protein (Ser235/236) antibody (clone 91B2, #4857, Cell Signaling Technology, Danvers, MA, USA), mouse monoclonal anti-C5 antibody (clone BB5.1, #HM1073, Hycult Biotech), mouse monoclonal anti-C3 antibody (clone 12E2, #NBP1-05140, Novus Biological, Centennial, CO, USA), mouse monoclonal anti-C5b-9 antibody (clone aE11, #M0777, Dako), rabbit polyclonal anti-tPA antibody (#10147-1-AP, Proteintech Group), rabbit polyclonal anti-plasminogen antibody (#GTX102877, Gene Tex), rabbit polyclonal anti-TNF-alpha antibody (#ab6671, abcam), rabbit polyclonal anti-IL-1beta antibody (#sc-7884, Santa Cruz Biotechnology), mouse polyclonal anti-COX-2 antibody (#160126, Cayman Chemical, Ann Arbor, MI, USA), and mouse monoclonal anti-smooth muscle α-actin antibody conjugated with Cy3 (clone 1A4, #C6198, Sigma-Aldrich, St. Louis, MI, USA).

Secondary antibodies used were as follows; Alexa Fluor 488-conjugated donkey anti-mouse IgG H&L antibody (#A21202, Thermo Fisher Scientific), Alexa Fluor 488-conjugated donkey anti-rabbit IgG H&L antibody (#A21206, Thermo Fisher Scientific), Alexa Fluor 594-conjugated donkey anti-mouse IgG H&L antibody (#A21203, Thermo Fisher Scientific), and Alexa Fluor 594-conjugated donkey anti-rabbit IgG H&L antibody (#A21207, Thermo Fisher Scientific).

### Digestion of a recombinant C5 protein by plasmin in a cell-free system

A recombinant C5 protein (100 μg/ml, lot: 083512, #A403, Quidel Corporation, San Diego, CA, USA) and each dose of recombinant Plasmin (0, 0.1, 0.33, 1, 3.3, 10, 33, and 100 μg/ml, lot: SLBV2192, #P1867, Sigma-Aldrich) were mixed and incubated for 1.5 h at 37 ℃ in a phosphate-buffered saline. The incubation mixture was then subjected to a western blot analysis using mouse monoclonal anti-human C5a/C5a des-Arg antibody (clone 2952, #HM2079, Hycult Biotech).

### Cell culture

NIH3T3 cell line, a mouse fibroblast-like cell line, was purchased from ATCC (#CRL-1658, Manassas, VA, USA) and cultured using Dulbecco’s Modified Eagle’s Medium (DMEM) (#044–29765, FUJIFILM Wako Pure Chemical Corporation, Osaka, Japan) supplied with 10% fetal bovine serum (FBS) (lot: 42F2102K, #10270-106, Thermo Fisher Scientific).

U-937 cell line, an established human cell line of histiocytic lymphoma origin, was purchased from ATCC (#CRL-1593.2) and cultured using a DMEM supplied with 10% FBS (lot: 42F2102K, #10270-106, Thermo Fisher Scientific). U-937 cell line was differentiated into macrophage-like cells by culturing with 10 μM Phorbol 12-myristate 13-acetate (PMA, lot: WDH4676, #162-23591, FUJIFILM Wako Pure Chemical Corporation) for 48 h.

### Quantitative real time-PCR (RT-PCR) analysis

NIH3T3 cells were treated with each dose of the selective mTOR (mammalian target of rapamycin) inhibitor Torin1 (IC_50_; 2.0 nM, lot: 3143151, #475991, Sigma-Aldrich) at the indicated dose and time period.

PMA-primed U937 cells were treated with each dose of recombinant C5a (lot: MBQ2518041, #2037-C5, R&D Systems, Minneapolis, MN, USA) at the indicated dose and time period. In some experiments, cells were pre-treated with the selective C5AR1 antagonist, W54011 (Ki = 2.2 nM, IC_50_; 1.6–3.1 nM, 300 nM, lot: H3118, #sc-203863, Santa Cruz Biotechnology).

The primary culture of intraperitoneal macrophage was treated with recombinant C5a (100 nM, 3 h, lot: P20210818251, #RPA388RA02, Cloud-Clone Corp., Katy, TX, USA) or lipopolysaccharide (LPS, 1 μg/ml, 3 h, lot: 074M4155V, #L3755, Sigma-Aldrich).

RNA purification from cultured cells and reverse transcription were done using a Rneasy Plus Mini Kit (#74136, QIAGEN) and a High-capacity cDNA Reverse Transcription Kit (#4368814, Life Technologies Corporation, Carlsbad, CA, USA) according to manufacturers’ instructions. For the quantification of gene expression, RT-PCR was performed on a LightCycler 480 (Roche, Basel, Switzerland) with a TB Green^®^ Premix Ex Taq™ II (Tli RnaseH Plus) (#RR820A, TAKARA BIO INC., Shiga, Japan) using the expression of β-actin (*ACTB* or *Actb*) as an internal control. For quantification, the second derivate maximum method was used for crossing point determination.

Primer sets used in the present experiment were as follows; forward 5′-AGCTTGGATATGTTGGGTTCC-3′ and reverse 5′-GGTAGTCTGGCCTTGAACTCC-3′ for *C5ar1*, forward 5′-CAGTAACAGTCCGCCTAGAAGC-3′ and reverse 5′-GCAAGTACTCTGTGTGGATTGG-3′ for *Actb* (mouse), forward 5′-TCAGCAATGAGTGACAGTTGG-3′ and reverse 5′-ATAGGCTGTTCCCATGTAGCC-3′ for *TNF*, forward 5′-ATTCAGCACAGGACTCTCTGG-3′ and reverse 5′-CAAGCTGGAATTTGAGTCTGC-3′ for *IL1B*, forward 5′-ACACCCTCTATCACTGGCATCC-3′ and reverse 5′-AACATTCCTACCACCAGCAACC-3′ for *PTGS2*, forward 5′-CATACTCCTGCTTGCTGATCC-3′ and reverse 5′-GATGCAGAAGGAGATCACTGC-3′ for *ACTB*, forward 5′-AGAGAGGAGGCTGACTTTCTCC-3′ and reverse 5′-GCATGGATCTCAAAGACAACC-3′ for *Tnf*, forward 5′-TCCAAGTTCTACCATGGTCTCC-3′ and reverse 5′-TCCCTGAAACCTTACACATCG-3′ for *Ptgs2*, forward 5′-GCAGTGCATACCACTTCAACC-3′ and reverse 5′-CTGATGGTCAAGATCCAGAGG-3′ for *Nos2*, and forward 5′-TTAGGAGAGCATTGGAAGTTGG-3′ and reverse 5′-CTTCTGGAGTTCCGTTTCTACC-3′ for *Il6*, forward 5′-CAGTAACAGTCCGCCTAGAAGC-3′ and reverse 5′-GCAAGTACTCTGTGTGGATTGG-3′ for *Actb* (rat)*.*

### Western blot analysis

NIH3T3 cells were pre-treated with Platelet-derived Growth Factor (PDGF)-BB (100 ng/ml, 30 min, lot: QXB1219032, #220-BB, R&D Systems), which was then cultured under the starved condition for 24 h or treated with Torin1 (500 nM, lot: 3143151, #475991, Sigma-Aldrich) for 1 h.

Whole cell lysate was prepared by a RIPA buffer (#R0278, Sigma-Aldrich) supplemented with proteinase and phosphatase inhibitors (#04693159001 and #4906837001, Roche). Protein concentration was, then, determined by a Bicinchoninic Acid (BCA) method (#23227, Pierce BCA Protein Assay Kit, Thermo Scientific). After SDS-PAGE (sodium dodecyl sulfate-poly-acrylamide gel electrophoresis), separated proteins were blotted to a PDVF membrane (Hybond-P, #10600058, GE healthcare, Buckinghamshire, UK) and blocked with an ECL plus blocking agent (#RPN2125, GE healthcare). The membranes were then incubated with primary antibodies followed by incubation with an anti-IgG antibody conjugated by horseradish peroxidase (anti-mouse IgG, #NA931V; anti-rabbit IgG, #NA934V, GE healthcare). Finally, the signal was detected by a chemiluminescent reagent (ECL Prime Western Blotting Detection System, #RPN2236, GE healthcare). Α-Tubulin was served as an internal control.

Primary antibodies used in Western Blot analysis were as follows; rabbit monoclonal anti-phospho-S6 ribosomal protein (Ser235/236) antibody (clone 91B2, #4857, Cell Signaling Technology), rabbit monoclonal anti-S6 ribosomal protein antibody (clone 5G10, #2217, Cell Signaling Technology), mouse monoclonal anti-α-tubulin antibody (clone DM1A, #T6199, Sigma-Aldrich).

### Statistics

Data are shown as mean ± s.e.m. Differences between the 2 groups were examined using the non-parametric Wilcoxon rank-sum test. Statistical comparisons between more than 2 groups were conducted using the Kruskal–Wallis test followed by the Steel test, or the Steel–Dwass test. A p value smaller than 0.05 was defined as statistically significant. Statistical analyses were performed with JMP software (version 14.0, SAS Institute, Cary, NC, USA).

## Results

### Induced expression of C5ar1 in ruptured or rupture-prone IA lesions of rats

**Figure 1:**
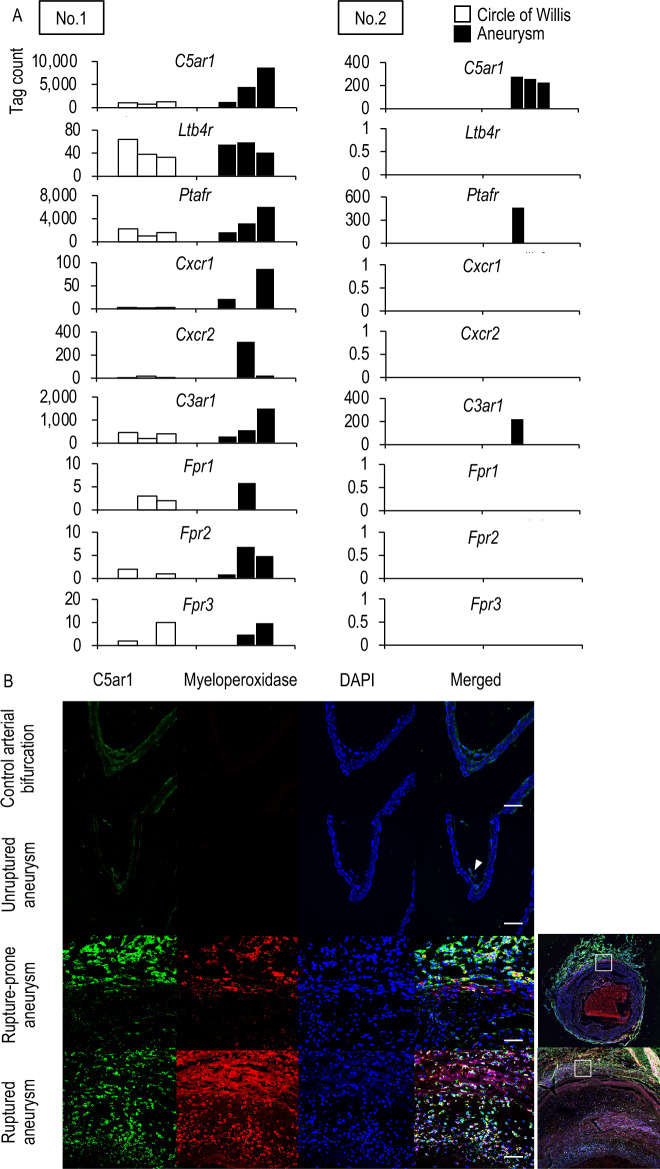
Induction of C5ar1 in rupture-prone or ruptured intracranial aneurysm lesions induced in rats. (**A**) The increase in tag count of *C5ar1* in specimens from intracranial aneurysms (IAs). Rupture-prone IA lesions, which have a high probability of rupture, or ruptured ones were induced in rats and the lesions (Aneurysm) accompanied with remaining portion of the circle of Willis (Circle of Willis) were harvested subjecting to comprehensive gene expression analyses. The tag count of each known factor mediating the migration of neutrophils like *C5ar1* from two-independent data sets is shown. The data presented in No. 1 (the left panel) were acquired from a previously deposited dataset (GEO accession: GSE161044) (n = 3 in each group), whereas that shown in No. 2 (the right panel) were from newly obtained samples in the present study (n = 5 in each group). Each bar indicates the actual tag count in each specimen. (**B**) Expression of C5ar1 in infiltrating neutrophils in IA lesions. IA lesions (Unruptured aneurysm which never ruptures, Rupture-prone aneurysm or Ruptured aneurysm) or control arterial bifurcations were harvested subjected to immunohistochemical analyses. The images of immunofluorescent staining of IA lesions for C5ar1 (green), a marker for neutrophils, Myeloperoxidase (red), nuclear staining by DAPI (blue), and merged images are shown. The arrowhead in the merged image of the unruptured aneurysm indicates the location of the aneurysm. Demagnified images of the rupture-prone and ruptured aneurysms are shown on the right. The squares in the demagnified images correspond to the area shown in the magnified images. Scale bar: 50 μm.

First, to narrow candidates to facilitate the migration of neutrophils into the site of rupture, which crucially contribute to the rupture of IAs^4^, the two independent comprehensive gene expression data sets previously (GEO accession: GSE161044; Fig. 1A, No. 1, n = 3 in each group)^4^ and newly obtained from the rat model of ruptured and rupture-prone IA lesions (Fig. 1A, No. 2, n = 5 in each group)^3,4,10^ were analyzed. Among known receptors mediating the migration of neutrophils, *C5ar1* was then picked up as a factor significantly up-regulated in ruptured and rupture-prone IA lesions in both data sets. The increase in the tag count of *C5ar1* was not observed in all of the rupture-prone IA samples, which is consistent with the fact that not all rupture-prone aneurysm rupture and that the histological features are diverse in aneurysm lesions^12,13^.

We next examined expression of C5ar1 protein in immunohistochemistry to corroborate the results from the comprehensive gene expression profile data. In control arterial walls or unruptured IA lesions which never rupture harvested from rats, the expression of C5ar1 was only weakly detected mainly on cells located at the most inner surface of arterial walls; endothelial cells and some other cells at the adventitia (Fig. 1B, Supplementary Fig. S1). Meanwhile, the expression of C5ar1 was clearly detected in both rupture-prone and ruptured IA lesions. Some signals for C5ar1 were co-localized with those for the neutrophil marker, Myeloperoxidase (Fig. 1B, Supplementary Fig. S1). Here noted that Myeloperoxidase-positive neutrophils express C5ar1 even before activated (Supplementary Fig. S2). Besides, the expression of C5ar1 in IA lesions was also detected at the adventitia negative for cell markers, such as Myeloperoxidase, CD 68 (i.e., a marker for macrophages), and smooth muscle α-actin (Supplementary Fig. S3). While there are no markers that are generally accepted as fibroblast-specific, mural cells that consist the majority of cellular components at the adventitia and form layers are known to be fibroblasts^14^. These findings thus suggest that the increased expression of C5ar1 in IA lesions originated from infiltrated neutrophils, which physiologically express C5ar1, and adventitial fibroblasts that induce C5ar1 in IA lesions.

### Up-regulated expression of *C5AR1* in human IA walls revealed by the comprehensive gene expression analyses and the protein expression of C5AR1 in human IA lesions

**Figure 2:**
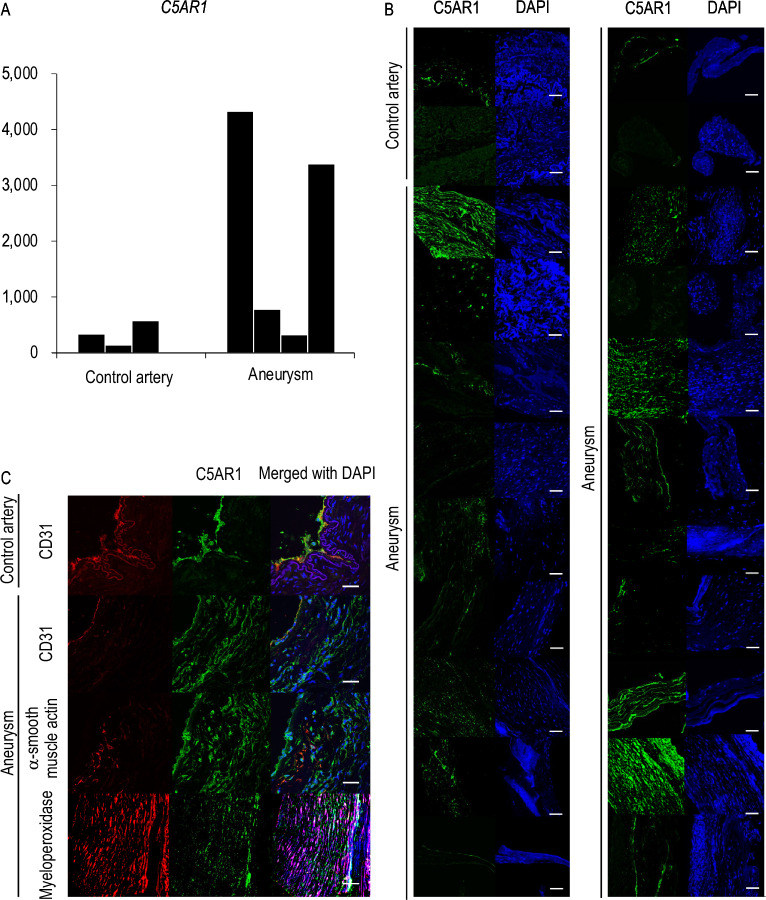
Up-regulation of C5AR1 in human intracranial aneurysm lesions. (**A**) The increase in tag count of *C5AR1* in human intracranial aneurysm (IA) lesions. IA lesions (Aneurysm, n = 4) or control arterial walls (Superficial temporal artery, n = 4) were harvested subjecting to comprehensive gene expression analyses. The tag count of *C5AR1* from acquired data sets is shown. Each bar indicates the actual tag count in each specimen. (**B**) Expression of C5AR1 in IA lesions. IA lesions (Aneurysm) or control arterial walls (Superficial temporal artery) were harvested subjected to immunohistochemical analyses. The images of immunofluorescent staining of control arterial walls or IA lesions for C5AR1 (green), nuclear staining by DAPI (blue), and merged images are shown. Scale bar: 50 μm. (**C**) Immunohistochemical analyses investigating cell types expressing C5AR1 in IA lesions. The images of immunofluorescent staining of control arterial walls (Superficial temporal artery) or IA lesions for C5AR1 (green), a marker for endothelial cells, CD31 (red), a marker for smooth muscle cells, α-smooth muscle actin (SMA, red), a marker for neutrophils, Myeloperoxidase (red), and merged images with nuclear staining by DAPI (blue) are shown. Scale bar: 50 μm.

By analyzing comprehensive gene expression analysis data from human specimens we previously obtained by RNA-sequencing (ID number #PRJNA553307 at http://www.ncbi.nlm.nih.gov/bioproject)^8^, the induction of *C5AR1* in IA lesions compared with those in control arterial walls (superior temporal artery) was reproduced also in human specimens (Fig. 2A) as in animal models (Fig. 1A). Here noted that the increase in the tag count of *C5AR1* is not necessarily observed in all of the human IA lesions analyzed, suggesting the heterogeneous nature of human IA lesions, which is consistent with a previously published study by Frösen et al.^13^.

We further performed immunohistochemistry to corroborate that the increase of mRNA expression indeed reflected protein expression in human lesions. We examined the immunohistochemistry of C5AR1 in 20 IA lesions and control arterial walls (superior temporal artery) obtained from a patient population different from that recruited in the RNA-seq experiment shown in Fig. 2A. The expression of C5AR1 was only weakly detected in control arterial walls (Fig. 2B, Supplementary Fig. S1), which was mainly on CD31-positive endothelial cells (Fig. 2C, Supplementary Fig. S1). Its expression could be consistently observed also in human IA walls (Fig. 2C, Supplementary Fig. S1), but in some lesions the expression in endothelial cells was reduced presumably due to damage and loss of endothelial cells (Fig. 2C, Supplementary Fig. S1). Although the expression of C5AR1 in human IA lesions varied among lesions, in some IA lesions, the signals for C5AR1 staining could be detected in some other types of cells than endothelial cells, including myeloperoxidase-positive neutrophils and cell marker-negative cells at the adventitia, which are presumably fibroblasts (Fig. 2B,C, Supplementary Fig. S1)^14^. The diversity of the staining patterns of C5AR1 in human IAs may be responsible for the heterogeneity of human IA lesions previously presented by Frösen et al.^13^.

These results combined together indicate the increase in C5AR1 expression is associated with infiltrated neutrophils, which physiologically express C5AR1, and adventitial fibroblasts that induce C5AR1 in IA lesions, which are consistent with findings in rat IA lesions.

### Potential mechanisms to induce C5ar1 expression in rat IA lesions

**Figure 3:**
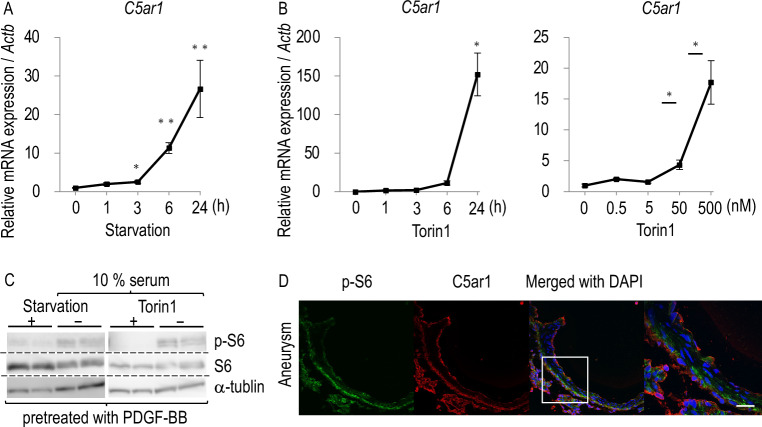
Potential role of mTOR pathway in the transcriptional induction of *C5ar1* in intracranial aneurysm lesions. (**A**) Up-regulation of *C5ar1* under starved condition in NIH3T3 cells. NIH 3T3 cells were cultured without serum (Starvation) for indicated period. Total RNA was then purified from treated cells subjected to quantitative RT-PCR analyses to examine the induction of *C5ar1*. Data represents as mean ± s.e.m (n = 8 in each group). Statistical analysis was done by the Kruskal–Wallis test followed by the Steel test, 0 h as the control. *p < 0.05, **p < 0.01. (**B**) Up-regulation of *C5ar1* in the selective mTOR inhibitor Torin1 treated-NIH3T3 cells. NIH 3T3 cells were cultured with Torin1 (250 nM, 0–24 h in the left panel; 0–500 nM, 24 h in the right panel). Total RNA was then purified from treated cells subjected to quantitative RT-PCR analyses to examine the induction of *C5ar1*. Data represents as mean ± s.e.m (n = 6 in each group). Statistical analysis was done by the Kruskal–Wallis test followed by the Steel test, 0 h as the control, in the left and by the Steel–Dwass test in the right panel. *p < 0.05. (**C**) Suppression of the phosphorylation in S6 under the starved condition or the treatment with Torin1. NIH3T3 cells pre-treated with PDGF-BB (100 ng/ml, 30 min) were cultured under the starved condition without serum (24 h) or the treatment with Torin1 (500 nM, 1 h). Total cell lysates were then prepared subjected to western blot analyses. The representative images from western blot analyses for phosphorylated form of S6 (p-S6) or S6 are shown. The immunoblot for α-tubulin were served as an internal control. (**D**) The images of immunofluorescent staining of rat intracranial aneurysm lesions for phosphorylated form of S6 (p-S6) (green), C5ar1 (red), and merged images with nuclear staining by DAPI (blue) are shown. The magnified image corresponding to the square in the left panel is shown in the right panel. Scale bar: 50 μm.

The molecular mechanisms to induce C5ar1 in IA lesions were next examined in in vitro experiments. In these experiments, we chose the NIH3T3 cell line, a mouse fibroblast-like cell line, because C5ar1 was thought to be induced in adventitial fibroblasts in IA lesions. Meanwhile, we did not select cell lines of inflammatory cells in these investigations because inflammatory cells physiologically express C5ar1 at a high level and thus are thought to be unsuitable for investigating the mechanisms of C5ar1 induction. The starvation achieved by depletion of serum from culture media significantly induced the transcriptional expression of C5*ar1* when applied to cells (Fig. 3A). Here, mammalian target of rapamycin (mTOR) signaling is representatively nutrition-sensitive and mediates growth factor signaling^15,16^. Because the selective mTOR inhibitor, Torin1^17^, could mimic the effect of the starvation on the expression of C5*ar1* at the dose- and the duration-dependent manner (Fig. 3B) and also on the phosphorylation of the downstream factor of mTOR signaling, p70 S6 kinase (S6), in western blot analyses in PDGF-BB pre-treated cells (Fig. 3C, Supplementary Fig. S4), the suppression of the mTOR signaling activity was indicated to be a possible mechanism regulating the induction of C5*ar1* in rupture-prone IA lesions. Furthermore, the immunohistochemistry for C5ar1 and the phosphorylated form of S6, which indicates the activation of the mTOR signaling cascade, was done in rupture-prone IAs of rats. The signals for C5ar1 and phosphorylated form of S6 were segregated (Fig. 3D), which is consistent with the results from in vitro experiments.

### Co-localization of C5AR1 and C5a/C5a des-arg

**Figure 4:**
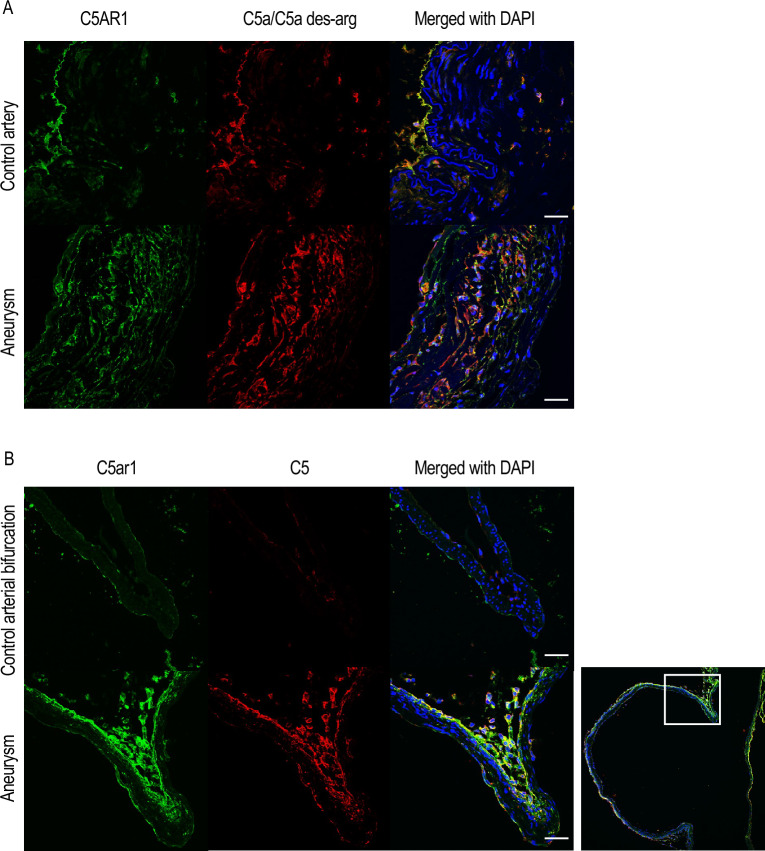
The co-expression of C5AR1 and its ligand C5a in intracranial aneurysm lesions. Specimens of intracranial aneurysm (IA) lesions (Aneurysm) or control arterial walls (Superficial temporal artery in (**A**), Anterior cerebral artery-olfactory artery bifurcation in (**B**)) from human cases (**A**) or rats (**B**) were harvested and examined in immunohistochemistry. The representative images of immunofluorescent staining of IA lesions for C5AR1 (green), C5a/C5a des-arg (red in (**A**)), C5 (red in (**B**)), and merged images with nuclear staining by DAPI (blue) are shown. A demagnified image of lower panels in (**B**) is shown on the right. The square in the demagnified image corresponds to the area shown in the magnified images. Scale bar: 50 μm.

Next, to clarify whether C5AR1 cascade may function in in vivo and thereby to support the potential involvement of C5AR1 signaling cascade in human IA pathogenesis, the expression of C5a, a ligand for C5AR1, was examined in immunohistochemistry. Intriguingly, most cells positive for C5AR1 staining were also positive for immunostaining using the antibody specifically recognizing its ligand C5a and also C5a des-arg, a derivative of C5a due to the deletion of arginine residue, in human IA lesions (Fig. 4A, Supplementary Fig. S1). As in human lesions, the signals for C5ar1 were well co-localized with those for C5 in rupture-prone IA lesions induced in rats (Fig. 4B, Supplementary Fig. S1). these findings indicate that the C5a–C5AR1 signaling may promote IA pathogenesis in humans and rats.

### Tissue-type plasminogen activator (tPA)-plasmin cascade as a putative upstream of C5a production in IA lesions of rats

**Figure 5:**
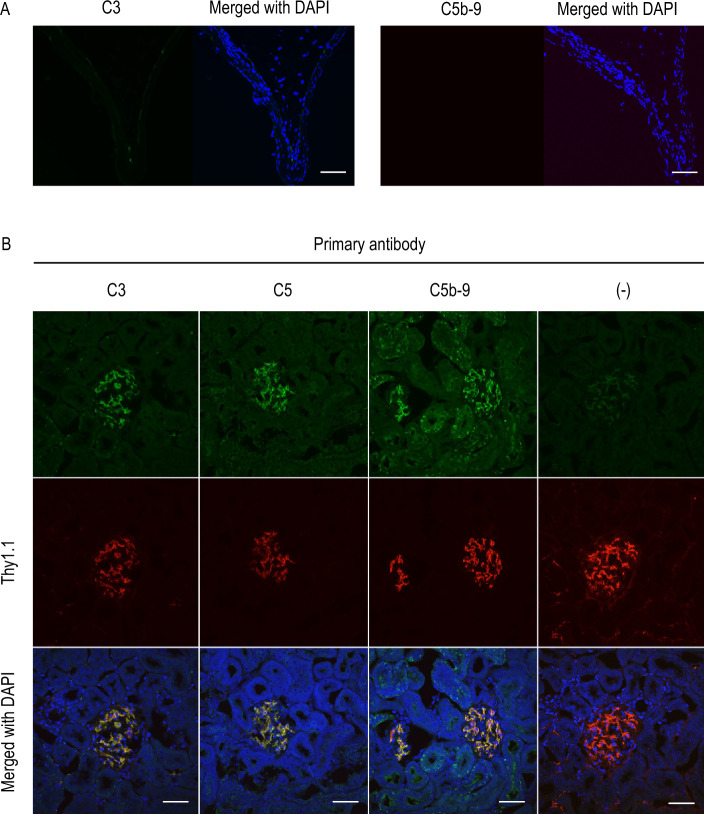
The absence of the deposition of complement C3 or C5b-9 complex in intracranial aneurysm lesions. (**A**) Intracranial aneurysm (IA) lesions induced in rats were harvested subjected to immunohistochemical analyses. The images of immunofluorescent staining of IA lesions for C3 (green in the left panel), C5b-9 (green in the right panel), and merged images with nuclear staining by DAPI (blue) are shown. Scale bar: 50 μm. (**B**) The deposition of complement C3, C5 or C5b-9 complex in the kidney from the anti-Thy1.1-induced glomerulonephritis model of rats. Glomerulonephritis was induced in rats by the injection of anti-Thy1.1 antibody, and the kidney was harvested subjected to immunohistochemical analyses. The images of immunofluorescent staining of kidney with glomerulonephritis for C3 (green), C5 (green), C5b-9 (green), Thy1.1 (red), and merged images with nuclear staining by DAPI (blue) are shown. The images from immunostaining without the primary antibodies are also shown as a negative control. Scale bar: 50 μm. This experiment was done to validate the specificity of antibodies used in (**A**).

The upstream signaling cascade to release C5a from C5 in situ was next examined because a factor releasing C5a could be a therapeutic target considering the potential role of the C5a–C5AR1 axis as a node to trigger the rupture of IA lesions. Whether the complement cascade functions to produce C5a was first examined in immunohistochemistry for C3 as an upstream of C5a production and the complement membrane attack complex (MAC) consisting of C5b-C9 as a downstream of the complement cascade. In control arterial walls, only the deposition of C5b-C9 complex could be observed at the adventitia (Supplementary Fig. S5). In IA lesions, neither the accumulation of C3 nor the deposition of C5b-C9 complex was present (Fig. 5), suggesting the presence of another cascade to release C5a from C5 in the lesions. Before we proceeded to investigate an alternative mechanism cleaving C5 to generate C5a other than complement cascades in rat IA lesions, we verified a positive control of the complement cascade activation in immunohistochemistry using the tissue section from anti-Thy-1.1-induced glomerulonephritis model, which is a representative disease caused by complement activation (Fig. 5B).

**Figure 6:**
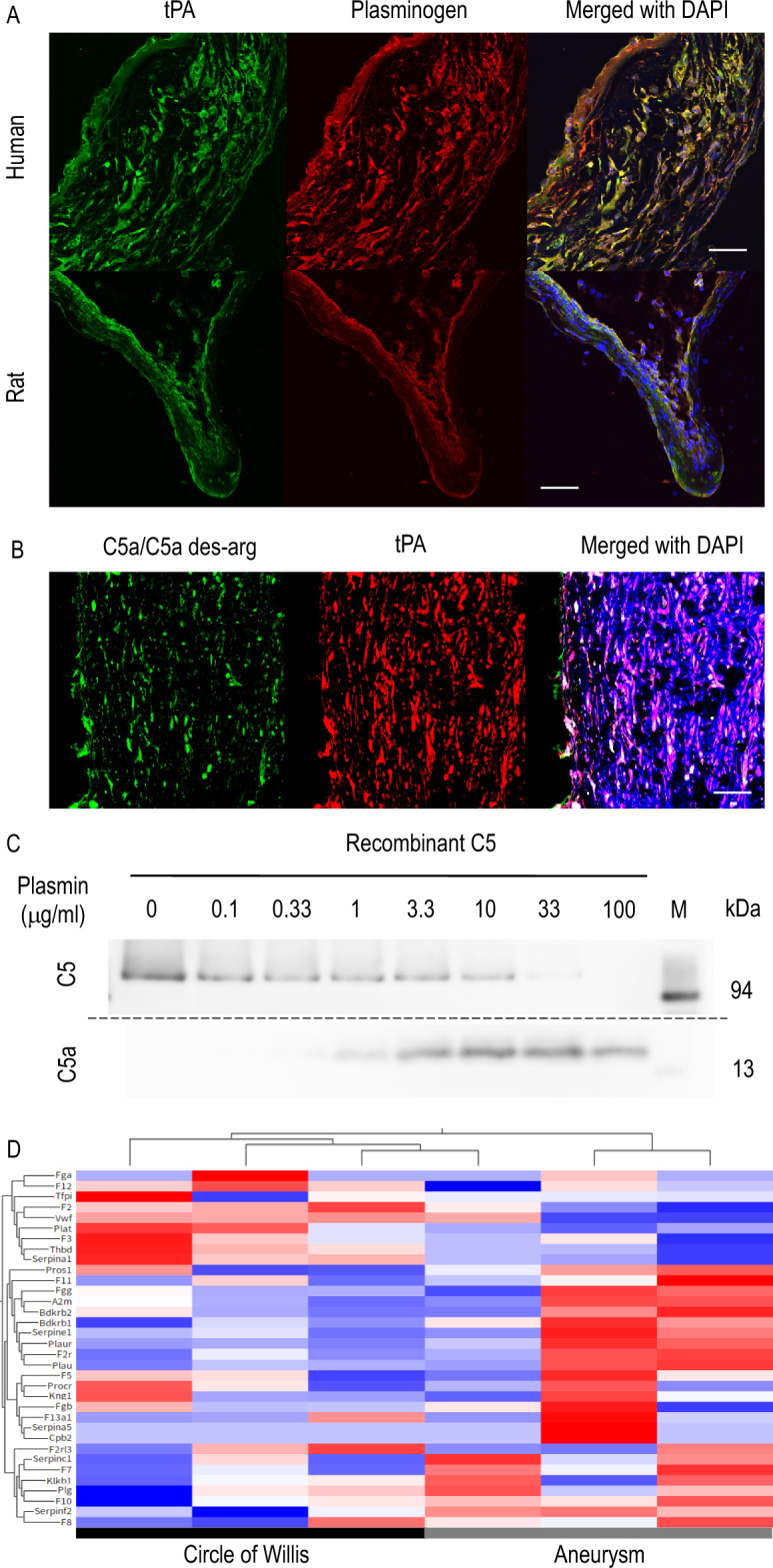
Tissue-type Plasminogen Activator as an up-stream factor to produce C5a from C5. (**A**) The expression of tissue-type Plasminogen Activator (tPA) and Plasminogen in intracranial aneurysm (IA) lesions. IA lesions from human cases or induced in rats were harvested subjected to immunohistochemical analyses. The images of immunofluorescent staining of IA lesions for tPA (green), Plasminogen (red), and merged images with nuclear staining by DAPI (blue) are shown. Scale bar: 50 μm. (**B**) Co-expression of tPA with C5a in IA lesions. Human IA lesions were harvested subjected to immunohistochemical analyses. The images of immunofluorescent staining of IA lesions for C5a/C5a des-arg which is the derivative of C5a (green), tPA (red), and merged images with nuclear staining by DAPI (blue) are shown. Scale bar: 50 μm. (**C**) Enzymatic cleavage of C5 into C5a by Plasmin. Recombinant C5 (100 μg/ml) was co-incubated with each dose of recombinant Plasmin as indicated (0–100 μg/ml) for 1.5 h in a cell-free system. The incubation mixture was subjected to western blot analyses. The representative images from the western blot analyses to detect C5 or C5a are shown. M; size marker. (**D**) The alternation in the coagulation-fibrinolysis system in IA lesions of rats. The heat map of genes related with the coagulation-fibrinolysis system from the comprehensive gene expression profile data set (GEO accession: GSE161044) is shown.

Referencing the previous report demonstrating the enzymatic cleavage of C5 to release C5a by Plasmin^18–20^, we next examine the presence of Plasminogen and tPA, which generates Plasmin from Plasminogen, in IA lesions. We then confirmed the co-localization of tPA and Plasminogen in same cells in lesions (Fig. 6A, Supplementary Fig. S1). Most signals for C5a/C5a des-arg was indeed co-localized with those for tPA (Fig. 6B, Supplementary Fig. S1). The Plasmin-dependent enzymatic cleavage of C5a from C5 was further confirmed in a cell-free system using recombinant C5 protein and the exogenous recombinant Plasmin (Fig. 6C, Supplementary Fig. S6). In addition, the role of tPA-Plasmin cascade, so-called coagulation-fibrinolysis cascade, to produce C5a in IA lesions as supported in immunohistochemistry was again indicated by comprehensive gene expression analysis, which demonstrated the alternation of the coagulation-fibrinolysis cascade in IA lesions (Fig. 6D).

### Effect of C5a on inflammatory responses in cultured cells

**Figure 7:**
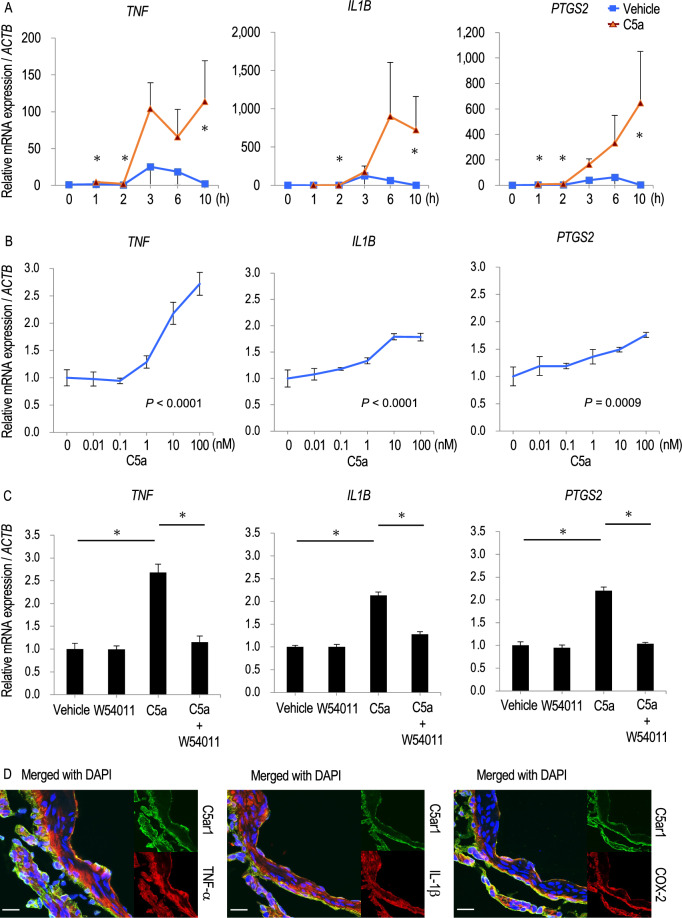
C5a–C5AR1 signaling cascade-dependent induction of pro-inflammatory factors. (**A**–**C**) The C5a–C5AR1 signaling cascade-dependent induction of pro-inflammatory genes in phorbol myristate acetate-primed cell line from human case with diffuse histiocytic lymphoma, U937 cell line. Cells were treated with recombinant C5a as indicated in the panels (0–10 h in (**A**), 0–100 nM in (**B**), 10 nM and 10 h in (**C**). Some cells were pre-treated with the selective C5AR1 inhibitor, W54011 (300 nM, 30 min) prior to the treatment with recombinant C5a. Total RNA was then purified from treated cells subjected to quantitative RT-PCR analyses to examine the induction of *TNF* (a gene for TNF-α), *IL1B* or *PTGS2* (a gene for COX-2; cyclooxygenase-2). Data represents as mean ± s.e.m ((**A**), n = 4; (**B**,**C**), n = 6). Statistical analysis was done by the Wilcoxon rank sum test in (**A**), the Kruskal–Wallis test followed by the Steel–Dwass test in (**B**,**C**). *p < 0.05. (**D**) Co-expression of C5a with pro-inflammatory factors in intracranial aneurysm (IA) lesions. IA lesions induced in rats were harvested subjected to immunohistochemical analyses. The images of immunofluorescent staining of IA lesions for C5ar1 (green), TNF-α (red), IL-1β (red), COX-2 (red), and merged images with nuclear staining by DAPI (blue) are shown. Scale bar: 50 μm.

The effect of C5a on inflammatory responses was examined using cultured PMA-primed U937 cells, a human macrophage-like cell line. We chose PMA-primed U937 cells in these experiments because inflammatory cells physiologically express C5AR1 and thus are suitable for investigating inflammatory responses to C5a. In this in vitro study, *TNF* (a gene for TNF-α), *IL1B* (IL-1β) and *PTGS2* (a gene for Cyclooxygenase-2; COX-2) were selected as representative genes reflecting inflammatory responses in situ because previous studies have clarified the crucial contribution of these factors to the pathogenesis of IAs^21–26^. The addition of recombinant C5a induced the expressions of *TNF*, *IL1B* and *PTGS2* at the dose- and the duration-dependent manner (Fig. 7A,B). Here, because the selective antagonist for C5AR1, W54011^27^, could interfere the effect of C5a on the pro-inflammatory gene expression to the level without stimulation (Fig. 7C), above effect of C5a was mediated by C5AR1. To evaluate the potential contribution of the C5a–C5AR1 signaling cascade to the expression of these pro-inflammatory factors in IA lesions, the expression of C5ar1, TNF-α, IL-1β and COX-2 in IA walls induced in rats was examined in immunohistochemistry. The signals for pro-inflammatory factors were well co-localized with ones for C5ar1 (Fig. 7D, Supplementary Fig. S1).

## Discussion

In the present study, we have identified that the C5a–C5AR1 axis may be a potential signaling cascade promoting the pathogenesis of IAs. The up-regulations of C5AR1/C5ar1 in human/rat IA lesions were indicated to stem from at least two different cellular components, i.e., infiltrated neutrophils, which physiologically express C5ar1, and adventitial fibroblasts that induce C5ar1 in IA lesions. Besides, the expression of C5AR1/C5ar1 and its ligand were co-localized in immunohistochemistry. Considering the role of neutrophils in the pathogenesis of IAs, the C5a–C5AR1 axis may function for recruiting neutrophils and provoking inflammation, which exaggerates the process leading to the rupture of IAs^4^. In addition, induction of C5AR1 occurred in mural cells such as fibroblasts, and thereby inflammation in IA lesions may be further boosted by the C5a–C5AR1 signaling in these mural cells. Therefore, the C5a–C5AR1 axis could be a therapeutic target to prevent the rupture of IAs. Intriguingly, induction of C5ar1 was demonstrated by starvation or the inhibition of mTOR signaling, which could occur in the microenvironment of inflammation by the increase in the energy demand.

In the present study, we have not detected the presence of the complement C3 and the complement complex C5b-9 in rat IA lesions, but instead identified the induced expression of tPA and Plasminogen in in vivo*.* Plasmin-dependent cleavage of C5 into C5a in a cell-free system was also confirmed. These findings suggested the production of C5a under the regulation by the coagulation-fibrinolysis system. Therefore, tPA may trigger C5a-related events, such as neutrophil recruitment and inflammatory responses, by converting Plasminogen into Plasmin and drive the pathogenesis leading to rupture of IAs. Thereby, tPA itself and also the machineries to induce tPA expression could be the key to distinguish rupture or rupture-prone lesions from many stable ones. The past study reported the induction of tPA in cultured endothelial cells upon turbulent flow loading^28^ and our study have consistently clarified the induced expression of tPA in endothelial cells in lesions. Considered with the results from computational fluid dynamic studies demonstrating the association of turbulent flow with aneurysm progression^29–33^, turbulent flow-induced tPA in addition to the inflammation may also mediate molecular events leading to rupture of lesions. These findings, including ours, indicating tPA-dependent progression of IAs are supported by a previously published study using an elastase-induced IA model in mice^34^, which showed that tPA was induced in IA lesions and promoted IA rupture^35^.

In the context of SAH, the involvement of C5a cascade in brain injury after the onset of SAH has been clarified^36^. Based on the findings from these studies, a randomized, open-label, Phase II clinical trial has been done to assess the effect of the antibody targeting C5, Eculizumab, on the production of C5a, adverse events, ischemic events or the clinical outcome^37^. Because of the nature of complement system as the innate system to rapidly response to external stimuli, this system might be similarly involved in microenvironment with inflammatory responses although the triggering factor to drive the complement system is presumably different between IAs and SAH. In the prevention of the enlargement or the rupture of intracranial aneurysms, the specific antibody for C5a might be applicable.

There are several limitations in the present study. First, the present study lacks experimental evidence supporting a causal relationship between the C5a–C5AR1 axis and the rupture of IA lesions. Second, the lack of C3 and C5b-9 in rat IA lesions in the present study was different from the findings in previous studies investigating human IA samples^38,39^. According to the present study, C5 in rat IAs was thought to be cleaved by alternative mechanisms other than complement cascades, such as by plasmin. Meanwhile, in humans, C5 may also be cleaved to generate C5a through complement cascades^38,39^. Although the findings in humans and rats were similar in that the C5AR-C5a axis may promote IA pathogenesis, the potential differences in the mechanisms underlying the cleavage of C5 between species were not addressed in this study. Third, differences in sex and surgical manipulations between the rupture-prone and non-rupture-prone models could not be addressed. The right external carotid artery and pterygopalatine artery are also ligated in addition to the left common carotid artery in the rupture-prone model. We could not answer about the potential impact of these factors on the observed difference in the C5a–C5ar1 axis between the two models.

In conclusion, in the present study, we have examined potential factors mediating the infiltration of neutrophils into IA lesions based on the recent experimental findings demonstrating the crucial role of neutrophils in the rupture of IAs. The C5a–C5ar1 axis was then identified as the candidate. Expression of C5AR1/C5ar1 is upregulated both in human and rat IA lesions, which originates from infiltrated neutrophils, which physiologically express C5AR1/C5ar1, and adventitial fibroblasts that induce C5AR1/C5ar1 in IA lesions. In IA lesions, C5a can be produced via cleavage by Plasmin in the coagulation-fibrinolysis pathway. We acknowledge that the most cardinal limitation in the present study is the lack of data showing the causal relationships between the up-regulation of C5AR1 and the progression of IAs. Nevertheless, our study demonstrates observational evidence indicating that the C5a–C5AR1 axis may be a therapeutic target to prevent the rupture of IAs.

### Supplementary Information

Supplementary Figure S1.

Supplementary Figure S2.

Supplementary Figure S3.

Supplementary Figure S4.

Supplementary Figure S5.

Supplementary Figure S6.

Supplementary Legends.

## Data Availability

All data generated or analysed during this study are included in this published article and its Supplementary Information files.
