# Supplementary figures and images for: C5a–C5AR1 axis as a potential trigger of the rupture of intracranial aneurysms
Source: Sci Rep. 2024 Feb 7;14:3105. doi: 10.1038/s41598-024-53651-7 (PMC10850553; doi:10.1038/s41598-024-53651-7)

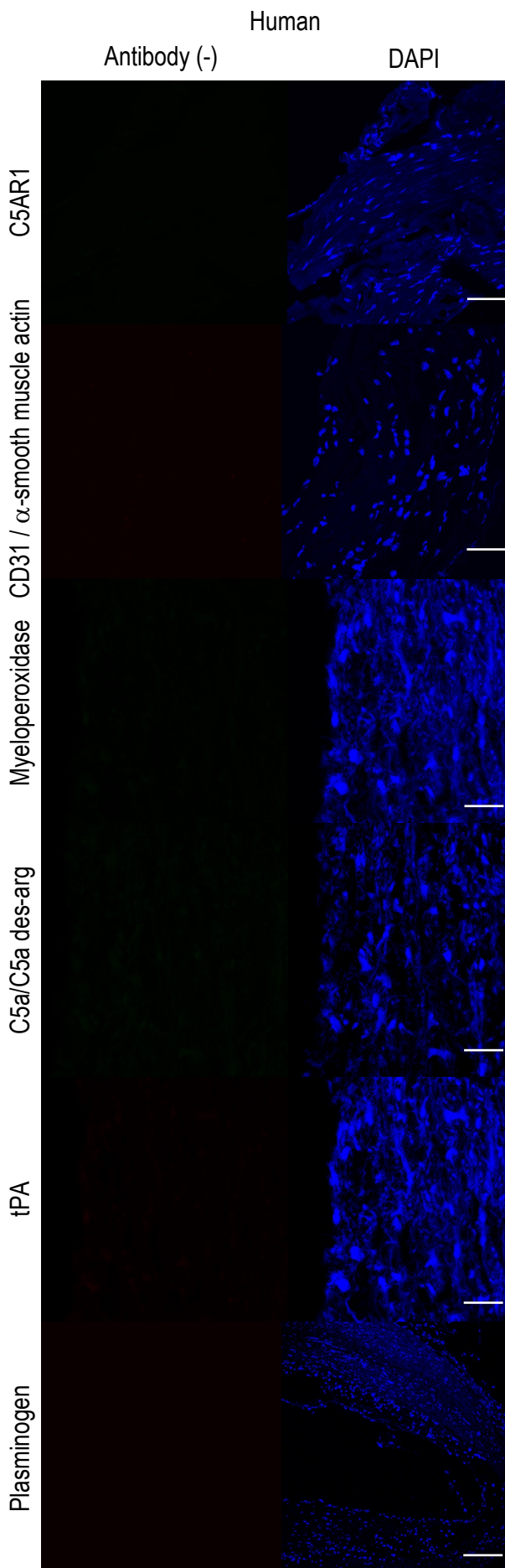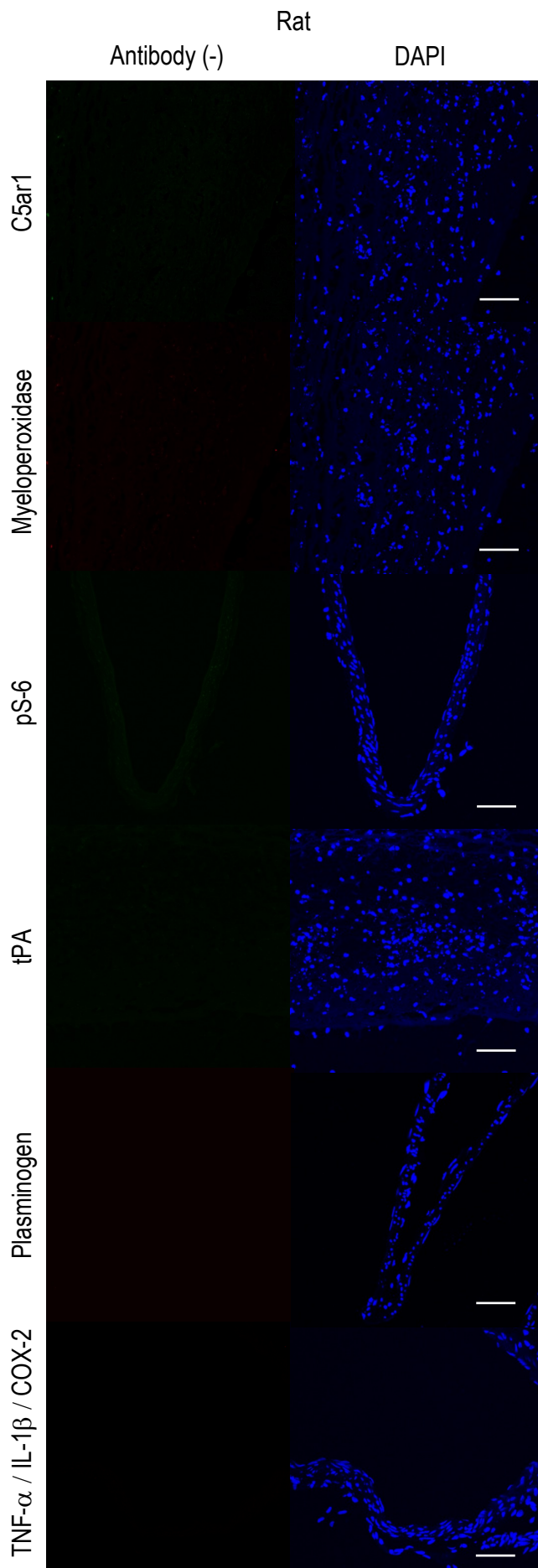

Supplement: Supplementary file 1 — Supplementary Figure S1. [file 41598_2024_53651_MOESM1_ESM.pdf]

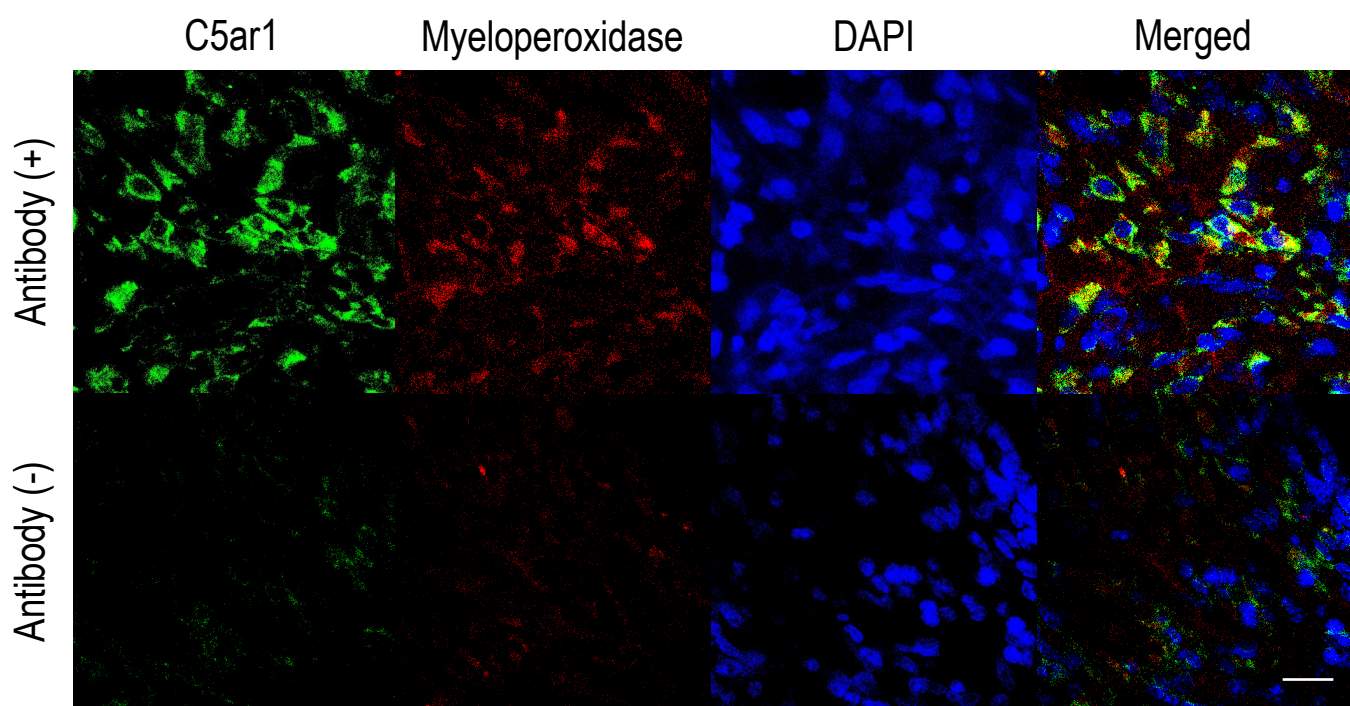

Supplement: Supplementary file 2 — Supplementary Figure S2. [file 41598_2024_53651_MOESM2_ESM.pdf]

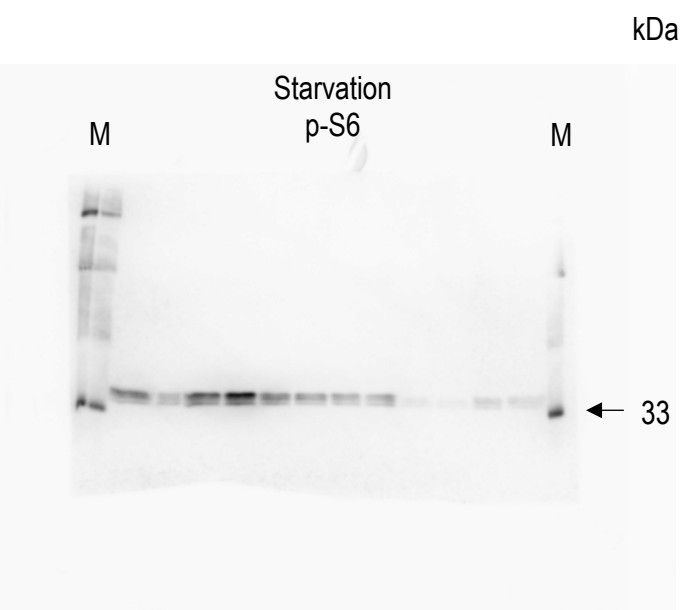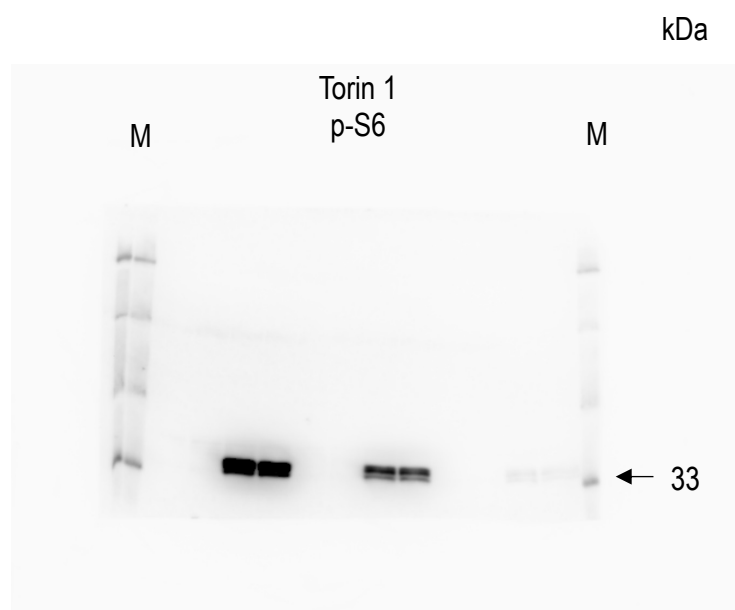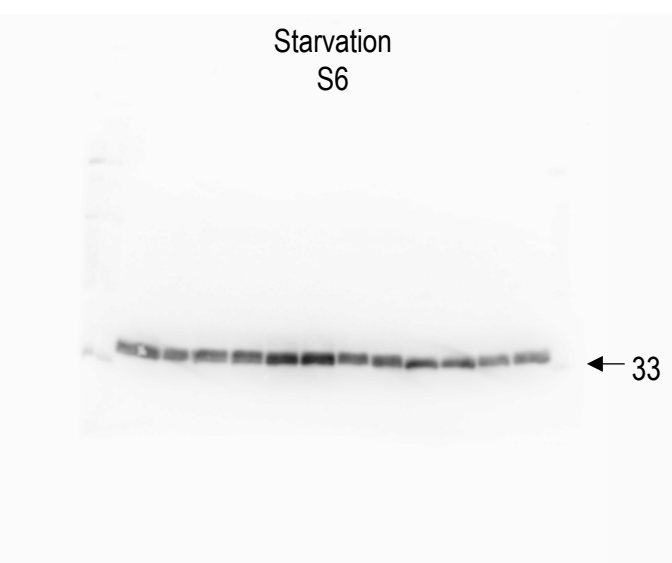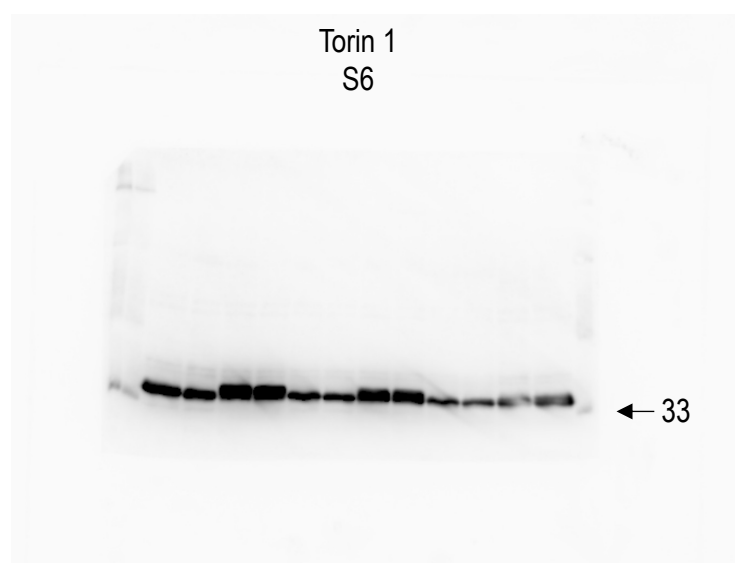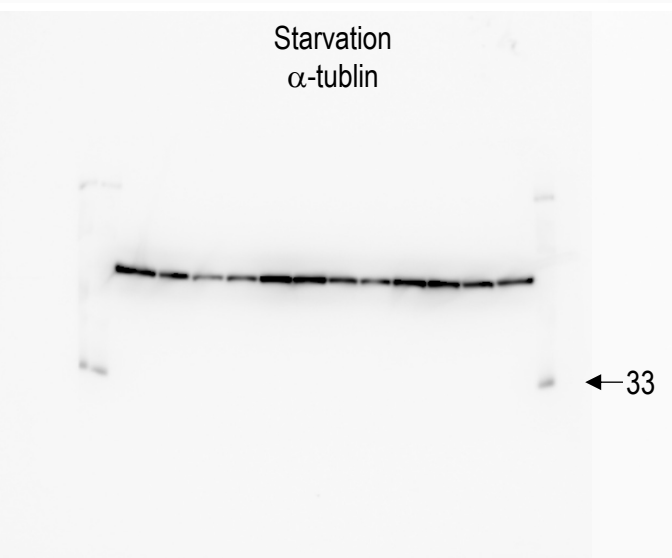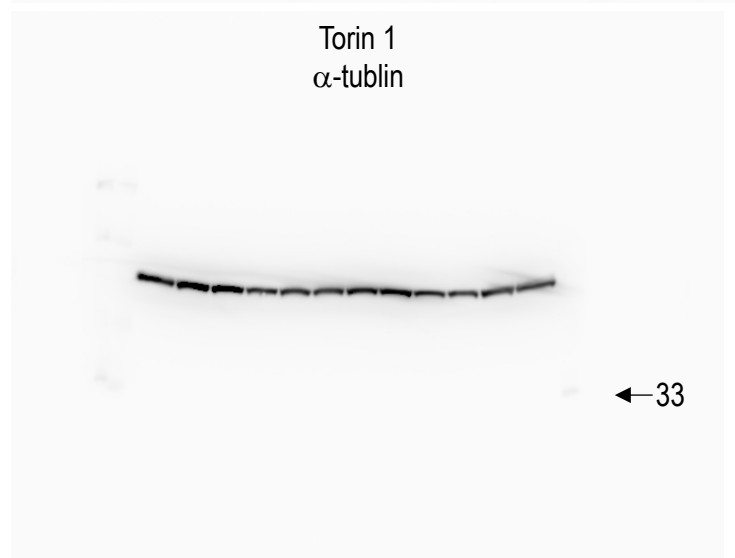

| 3h         |   | 9h |   | 24h |   |
|------------|---|----|---|-----|---|
| +          | - | +  | - | +   | - |
| Starvation |   |    |   |     |   |

Time course

| 3h     |   | 9h |   | 24h |   |
|--------|---|----|---|-----|---|
| +      | - | +  | - | +   | - |
| Torin1 |   |    |   |     |   |

Time course

Supplement: Supplementary file 4 — Supplementary Figure S4. [file 41598_2024_53651_MOESM4_ESM.pdf]

Merged with DAPI

C3

C5b-9

Antibody (-)

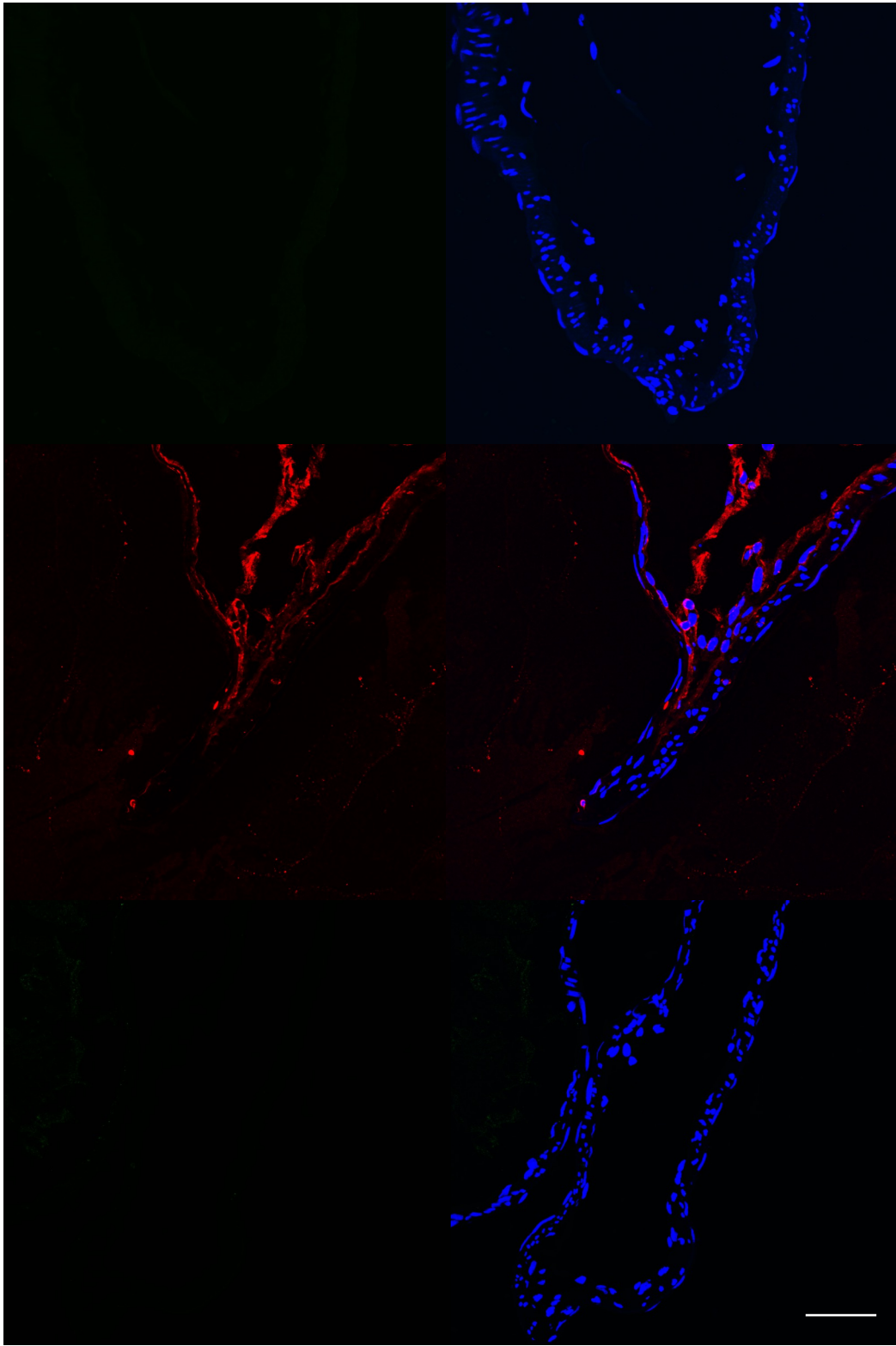

Supplement: Supplementary file 5 — Supplementary Figure S5. [file 41598_2024_53651_MOESM5_ESM.pdf]

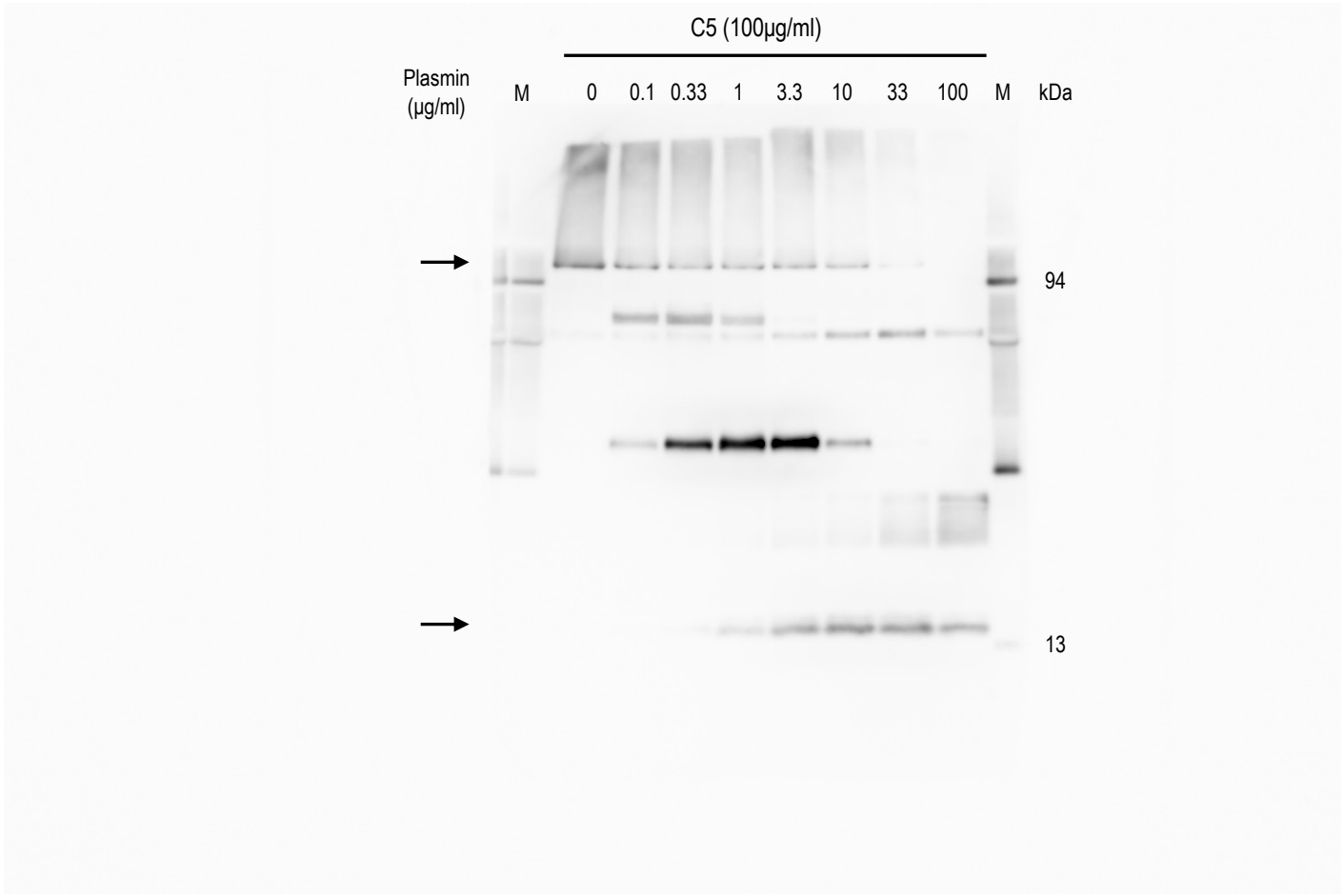

Supplement: Supplementary file 6 — Supplementary Figure S6. [file 41598_2024_53651_MOESM6_ESM.pdf]
