# Supplementary material for: C5a–C5AR1 axis as a potential trigger of the rupture of intracranial aneurysms
Source: Sci Rep. 2024 Feb 7;14:3105. doi: 10.1038/s41598-024-53651-7 (PMC10850553; doi:10.1038/s41598-024-53651-7)
Supplement: Supplementary file 3 — Supplementary Figure S3. [file 41598_2024_53651_MOESM3_ESM.pdf]

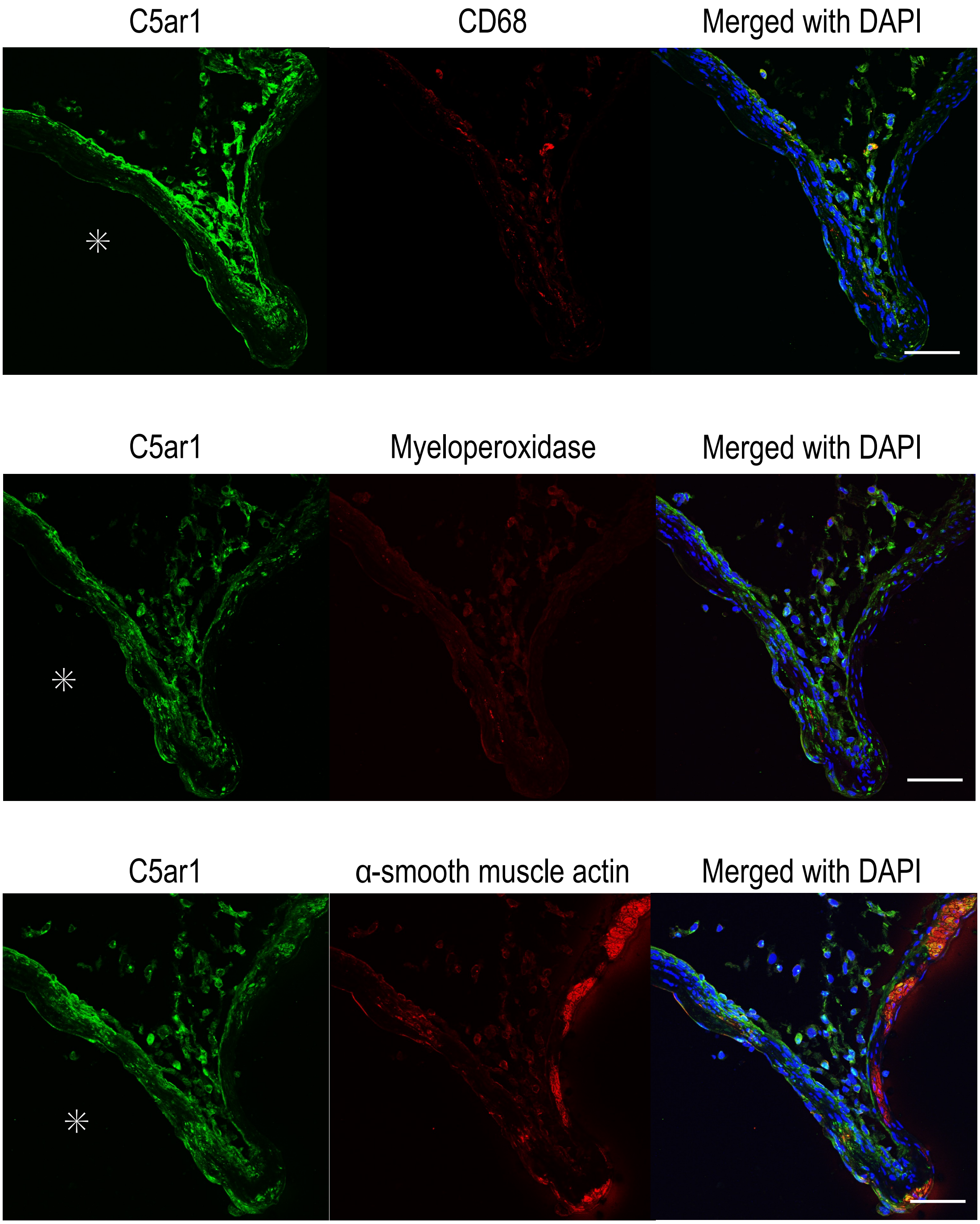

C5ar1
CD68
Merged with DAPI
C5ar1
Myeloperoxidase
Merged with DAPI
C5ar1
α-smooth muscle actin
Merged with DAPI
