# Supplementary material for: C5a–C5AR1 axis as a potential trigger of the rupture of intracranial aneurysms
Source: Sci Rep. 2024 Feb 7;14:3105. doi: 10.1038/s41598-024-53651-7 (PMC10850553; doi:10.1038/s41598-024-53651-7)
Supplement: Supplementary file 7 — Supplementary Legends. [file 41598_2024_53651_MOESM7_ESM.docx]

**Figure Legends**

**Supplementary Fig. S1. The negative control experiments in immunohistochemistry.**

The representative images of immunohistochemistry without a primary antibody as indicated are shown. Scale bar: 50 μm.

**Supplementary Fig. S2. Expression of C5ar1 on neutrophils in the spleen.**

The spleen was harvested and subjected to immunohistochemical analyses. The images of immunofluorescent staining for C5ar1 (green), the marker for neutrophils, Myeloperoxidase (red), nuclear staining by DAPI (blue), and merged images are shown. Immunohistochemistry without a primary antibody is served as an internal control. Scale bar: 10 μm.

**Supplementary Fig. S3.** Expression of C5ar1 in rupture-prone IA lesions of rats. The images of immunofluorescent staining of IA lesions for C5ar1 (green), a marker for macrophages, CD68 (red), a marker for neutrophils, Myeloperoxidase (red), a marker for neutrophils, Myeloperoxidase (red) , a marker for smooth muscle cells, α-smooth muscle actin (SMA, red), nuclear staining by DAPI (blue), and merged images are shown. The asterisks in the left panels indicate the luminal side of the aneurysm. Scale bar: 50 μm.

**Supplementary Fig. S4. The effect of the starved condition or Torin1 on the phosphorylation of S6.**

NIH3T3 cells pre-treated with PDGF-BB (100 ng/ml, 30 min) were cultured under the starved condition without serum (3, 9, and 24 h) or the treatment with Torin1 (300 nM, 30 min; 3, 9, and 24 h). Total cell lysates were prepared subjected to western blot analyses. The raw images of full membranes from western blot analyses for phosphorylated form of S6 (p-S6), S6, or α-tubulin used in Fig. 3C are shown. Information about the presence or absence of starved condition or the treatment with torin1 and the time course of the experimental data is presented at the bottom. M; size marker.

**Supplementary Fig. S5. Accumulation or deposition of complements in control arterial walls.**

Anterior cerebral-olfactory artery bifurcation in rats were harvested and subjected to immunohistochemical analyses. The images of immunofluorescent staining of IA lesions for C3 (red in the upper panel), C5b-9 (red in the lower panel), and merged images with nuclear staining by DAPI (blue) are shown. Immunohistochemistry without a primary antibody is served as an internal control. Scale bar: 50 μm.

**Supplementary Fig. S6. Enzymatic cleavage of C5 into C5a by Plasmin.**

Recombinant C5 (100 ug/ml) was co-incubated with each dose of recombinant Plasmin as indicated (0~100 ug/ml) for 1.5 h in a cell-free system. The incubation mix was subjected to a western blot analysis. The image of the full membrane used in Fig. 6C is shown. The arrows indicate the part of the image stripped for Fig. 6C. M; size marker.
